# Single-Atom Catalysts in Environmental Engineering: Progress, Outlook and Challenges

**DOI:** 10.3390/molecules28093865

**Published:** 2023-05-04

**Authors:** Zhe Li, Rongrong Hong, Zhuoyi Zhang, Haiqiang Wang, Xuanhao Wu, Zhongbiao Wu

**Affiliations:** Department of Environmental Engineering, Zhejiang University, Hangzhou 310058, China; 22214036@zju.edu.cn (Z.L.); 22214030@zju.edu.cn (R.H.); 22214096@zju.edu.cn (Z.Z.); haiqiangwang@zju.edu.cn (H.W.)

**Keywords:** single-atom catalysts, VOCs treatment, NO_x_ reduction, CO_2_ reduction, CO oxidation, fenton-like processes, hydrodehalogenation, nitrate and nitrite reduction

## Abstract

Recently, single-atom catalysts (SACs) have attracted wide attention in the field of environmental engineering. Compared with their nanoparticle counterparts, SACs possess high atomic efficiency, unique catalytic activity, and selectivity. This review summarizes recent studies on the environmental remediation applications of SACs in (1) gaseous: volatile organic compounds (VOCs) treatment, NO_x_ reduction, CO_2_ reduction, and CO oxidation; (2) aqueous: Fenton-like advanced oxidation processes (AOPs), hydrodehalogenation, and nitrate/nitrite reduction. We present the treatment activities and reaction mechanisms of various SACs and propose challenges and future opportunities. We believe that this review will provide constructive inspiration and direction for future SAC research in environmental engineering.

## 1. Introduction

Large amounts of pollutants are discharged into the environment as a result of economic growth, leaving serious pollution problems that require urgent treatment. Compared to traditional physical adsorption or biological treatments, chemical catalysis is considered an effective approach to quickly degrade pollutants [1], with less generation of secondary solid waste or sludge. Developing appropriate catalysts that can not only efficiently eliminate pollutants but also operate stably and sustainably is of great importance [2].

Conventional heterogeneous catalysts are typically designed on a nanometer scale. However, the atomic utilization of nanoparticles (NPs) is limited because only the outmost layer of atoms participates in the surface catalytic reaction [3], which hinders the further improvement of catalytic activity. Moreover, noble metal catalysts containing costly Pd, Pt, Au, Ru, etc. are required to achieve higher atomic efficiency to obtain economic benefits. To solve these issues, researchers have devoted themselves to decreasing the size of nanocatalysts to maximize the exposure of active surface sites, meanwhile achieving additional benefits such as quantum size effects [4,5] and unsaturated coordination [6].

The idea of single-atom catalysts (SACs) was first proposed by Zhang and coworkers [7] in 2011, which describes a type of catalyst reaching the theoretical size limit of “single-atom”. Compared to bulk nanocatalysts, single-atom catalysts possess several advantages. From the perspective of catalyst structure, the sufficient interactions generated by the chemical bond between the metal and the support provide higher numbers of interfaces and active sites for the catalytic reaction [8,9,10,11]. The unsaturated coordination facilitates the adsorption of pollutants on the SAC site and dynamic electron transport, contributing to a better redox reaction [7,10,12]. The strong metal-support bonding also prevents the aggregation of atoms [13,14] and the environmental risk of metal leaching [15]. With close to 100% atomic efficiency, the metal loading greatly decreases to achieve a similar degradation capacity as nanocatalysts, further reducing the cost of the catalyst.

**Figure 1:**
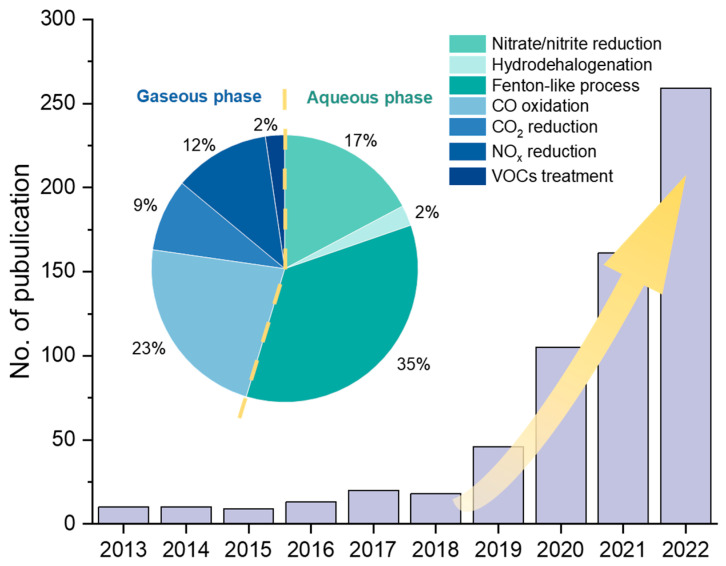
Number of publications of SAC research in environmental remediation applications and the proportion in various fields in the last decade. The publication data from 2013 to 2022 was collected from the Web of Science in April 2023.

In the field of environmental engineering, remarkable progress has been made in SAC research (Figure 1), particularly involving CO oxidation [7,16,17], CO_2_ reduction [18,19,20,21], NO_x_ degradation [22,23,24], volatile organic compounds (VOCs) degradation [23,25], aqueous advanced oxidation processes (AOPs) [26,27,28], hydrodehalogenation [29], nitrate reduction [30,31], etc. To date, there are few systematic summaries and reviews of SACs’ applications in environmental engineering. Therefore, in this review, we summarize recent studies on SAC applications in gaseous and aqueous pollution control, respectively, focusing on treatment efficiencies and reaction mechanisms. We further propose suggestions on the synthesis strategies and discuss the challenges and directions for future SAC research in the environmental engineering field. 

## 2. Progress of SACs in Gaseous Pollution Control

### 2.1. VOC Treatments

Volatile organic compounds (VOCs) are ubiquitous air pollutants that are mainly emitted from fossil fuel combustion, transportation, and industrial and household activities [32,33]. There are a wide variety of VOCs, including non-methane hydrocarbons (e.g., alkanes, aromatics), oxygen-containing organic compounds (e.g., aldehydes, ketones, alcohols, ethers), halogenated hydrocarbons, nitrogen- and sulfur-containing compounds, etc. The outdoor VOCs are important precursors of photochemical smog [34], and the indoor VOCs are detrimental to human health, with the probability of causing cancer [35]. The Chinese Fourteenth Five-Year Plan (2021–2025) [36] proposes to further advance the comprehensive management of VOC emissions and requires a more than 10% reduction of the total VOC emissions compared to 2020. Given the adverse impacts of VOCs on the environment and the new legislation in place, it is critical to develop efficient and applicable technologies to reduce VOC emissions. Catalytic oxidation is one of the most promising approaches due to its desirable features, such as high efficiency and energy savings [37], among traditional VOC abatement technologies including adsorption, condensation, thermal incineration, and biological degradation [33].

**Figure 2:**
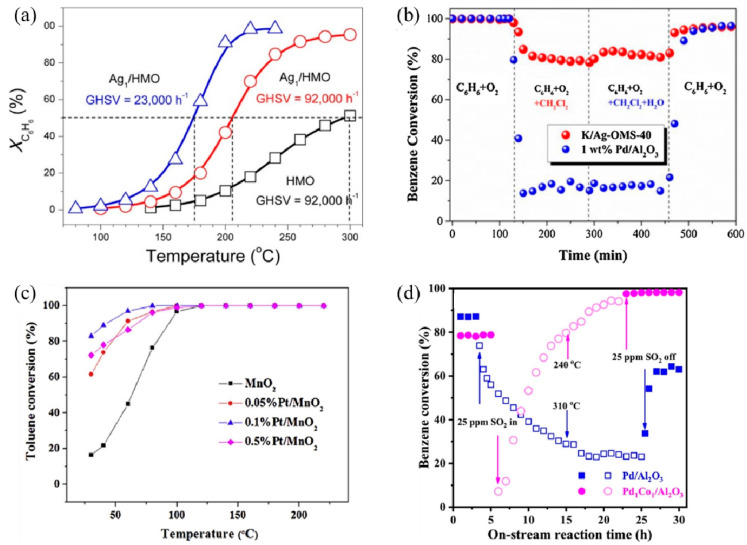
Catalytic performance of different SACs for several VOC treatments. (**a**) Conversion of benzene (*X*_C6H6_) as a function of temperature over Ag_1_/HMO and HMO at different GHSVs Reaction conditions: benzene, 200 ppm; O_2_, 20% and balanced by N_2_; flow rate, 100 mL min^−1^. Copyright 2017, American Chemical Society [40]. (**b**) Comparison of C_6_H_6_ conversion between K/Ag–OMS–40 (GHSV = 45,000 h^−1^) and 1 wt% Pd/Al_2_O_3_ (GHSV = 40,000 h^−1^) and stability test in terms of chlorine and moisture tolerance at a temperature of 300 °C. Copyright 2018, Elsevier B.V. [41]. (**c**) Temperature-dependent toluene conversion by MnO_2_ and Pt-deposited MnO_2_ catalysts (toluene inlet concentration: 10 ppm, 21% O_2_, N_2_ as balance gas, GHSV: 60 L g^−1^ h^−1^). Copyright 2019, Elsevier B.V. [43]. (**d**) Benzene conversion as a function of on-stream reaction time in the presence or absence of SO_2_ over the as-obtained samples. Copyright 2021, Elsevier B.V. [47].

SACs can maximize atomic efficiency, minimize the usage of noble metals, and achieve high activity and selectivity [9,38,39], thus attracting much attention in VOC treatments. In recent years, several noble metal SACs have been developed for VOC catalytic oxidation and showed superior performance compared to their nanoparticle counterparts, including Ag [40,41], Au [23,42], Pt [43,44,45], and Pd [46]. The single-atom Ag based on nanostructured hollandite manganese oxide (Ag_1_/HMO) [40] prepared by a thermal diffusion method achieved 100% conversion of benzene oxidation at 220 °C at a GHSV of 23,000 h^−1^ (Figure 2a). The isolated Ag adatoms possessed an excellent ability to activate lattice oxygen and gaseous O_2_ owing to their upshifted 4d orbitals. Comparably, the Ag atoms incorporated into cryptomelane-type manganese oxide (K/Ag–OMS-40) [41] showed higher benzene conversion, excellent stability, and enhanced tolerance to chlorine poisoning and moisture than 1 wt% Pd/Al_2_O_3_ (Figure 2b) [40,41]. The increased number of Mn octahedral defects and newly formed Ag–O–Mn interaction entities accelerated charge transfer [41], facilitating the benzene conversion. 

Au SACs also play an important role in low-temperature HCHO oxidation. Au_1_/α-MnO_2_ [42] and Au_1_/CeO_2_ [23] both exhibited remarkable activity and stability as the doped Au facilitates the formation of oxygen vacancies, active oxygen species, and charged Au species as active sites [23,42]. Au_1_/α-MnO_2_ completely degraded the 500 ppm HCHO pollutant stream at 75 °C, with a WHSV of 6 L g^−1^ h^−1^. As for Au_1_/CeO_2_, among different CeO_2_ morphologies, CeO_2_ rod-supported Au (Au/r–CeO_2_) as an optimal catalyst successfully achieved complete mineralization of HCHO at 85 °C. Additionally, Pt SACs exhibit good VOC catalytic performance as well. For example, the Pt_1_/MnO_2_ [43] synthesized via hydrothermal process achieved 100% conversion of indoor-level toluene at ambient temperature due to the formation of surface active oxygen species, including hydroxyl radicals (^•^OH) (Figure 2c). Chen et al. [45] screened out 0.47 wt% Pt_1_/Mn–TiO_2_ as the optimal catalyst with extraordinary activity and acceptable cost, which completely eliminated HCHO (100 ppm) at room temperature.

Moreover, non-noble metal SACs are also applied in VOC catalytic oxidation. An Al SAC-doped graphene was proposed through density functional theory (DFT) calculations for the catalytic oxidation of HCHO at room temperature [48]. Through a pathway of HCHO→HCOOH→CO→CO_2_, the energy barriers for breaking the C–H bond in HCHO and the C–O bond in HCOOH were both 0.82 eV, serving as the kinetic limiting steps. A bimetal single-atom Pd_1_Co_1_/Al_2_O_3_ catalyst with double active sites showed enhanced catalytic performance and sulfur resistance for benzene oxidation, over which a 90% benzene conversion was realized at 256 °C, and a gradual recovery of activity after the introduction of 25 ppm SO_2_ was observed (Figure 2d) [47]. In situ temperature-programmed experiments, in situ diffuse reflectance infrared Fourier transform spectroscopy (DRIFTS), and X-ray absorption fine structure (XAFS) characterizations demonstrated the synergistic behaviors between Co_1_ and Pd_1_ sites. The O = Co = O species formed rapidly on the Co_1_ site to activate oxygen, while benzene selectively tended to adsorb on the Pd_1_ site. According to previous studies, due to the *π*-bond in the benzene molecule, a parallel or flat configuration is formed on the close-packed transition metal surfaces [49]. The Pd_1_ and Co_1_ double active sites inhibited the competitive adsorption between benzene and oxygen, thus enhancing the reactivity. Meanwhile, the PdO–SO_3_ complex formed after the addition of SO_2_ was decomposed into PdO, reactive oxygen species (ROS), and aluminum sulfite at low temperatures, while ROS and PdO sites continued to participate in the reaction, leading to high sulfur resistance. 

### 2.2. CO Oxidation

**Figure 3:**
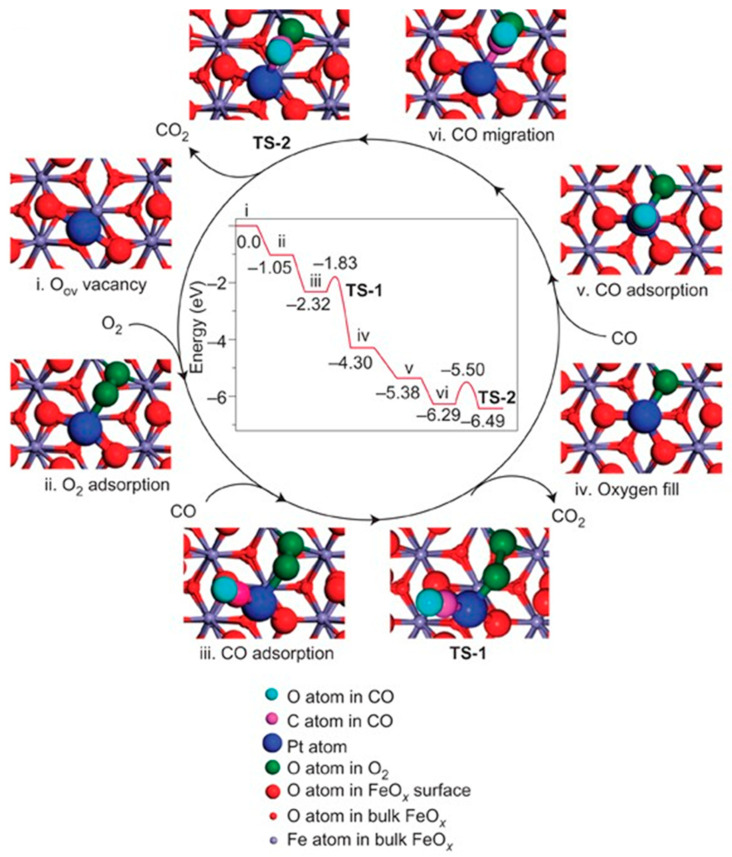
The proposed reaction pathways and energy profile (in *eV*) for CO oxidation on the single-atom catalyst Pt_1_/FeO_x_. Copyright 2011, Nature Publishing Group [7].

Carbon monoxide (CO), an odorless and toxic gas due to its high affinity with hemoglobin in the blood [50], widely exists in the exhaust of the automobile and multiple industrial processes [51]. Over the past few decades, CO oxidation methods have been investigated to deal with CO emissions [52]. To overcome the low activity, poor stability, and high cost of current catalysts [53,54], numerous SACs have attracted considerable attention in CO oxidation both experimentally and theoretically, including noble metal catalysts (Pt (Figure 3) [7], Au [55,56,57,58], Pd [59]), non-noble metal catalysts (Fe [60], Co [61], Ni [62]), and metal-free catalysts (Si [63], B and S [64]).

Gold nanocatalysts have shown outstanding performance in low-temperature CO oxidation [55,56,57,58]. As large Au particles are inert for O_2_ activation, it is important to reduce the particle size. The Au_1_/FeO_x_ SAC [65] with an extremely low loading of 0.015 wt% achieved a high turnover frequency (TOF) of 0.49 s^−1^ at 24 °C, which was almost 10 times higher than that of the Au/Fe_2_O_3_ catalyst with a loading of 4.4 wt% at 27 °C [66]. It also achieved higher sintering resistance than Au nanocatalysts. By means of extensive first-principles calculations [67], undergoing a local reconstruction, single-atom Au in Ni- and Cu-doped Au@TiO_2_ were atomically deposited at oxygen vacancies on the TiO_2_ and formed stable “O–Au–O” species. The oxidation states of the Au cation SAC can be tuned via substrate doping with a transition metal to further improve the O_2_ activation. The highly oxidized Au single atom showed magnetism and promoted activity and stability for O_2_ activation and CO oxidation. 

The high cost of noble metals can be an obstacle to their practical application, so it is necessary to exploit non-noble and non-mental catalysts. It was elucidated theoretically that the Fe_1_/C_2_N monolayer can catalyze CO oxidation via a two-step mechanism due to the localized metal 3*d* orbitals near the Fermi level [60]. The mechanism of CO oxidation mediated by single Cu atom-doped clusters CuAl_4_O_7–9_^−^ was experimentally identified, and CO was found to be crucial to stabilizing Cu in CuAl_4_O_9_^−^ around the +1 oxidation state [68]. Moreover, the single-atom Si can be stably embedded into the center of N_4_ in graphene (Si–GN_4_) and effectively regulate the electronic structure of the GN_4_ system, enhancing O_2_ adsorption [63]. According to the first-principles method, Si–GN_4_ had excellent stability and catalytic activity at high temperatures. The steps of the complete CO oxidation on Si SAC were as follows: CO + O_2_→OOCO→CO_2_ + O_ads_, 0.57 eV, followed by a second reaction: CO + O_ads_→CO_2_, 0.72 eV. Lee and Yan et al. [64] reported the CO oxidation mechanism on a sulfur-doped hexagonal boron nitride (*h*–*BN*) non-mental catalyst. The sulfur-doped *h–BN* accelerated the oxidation of CO by reducing the energy barrier of O_2_ chemisorption.

### 2.3. NO and N_2_O Reduction

High volumes of NO_x_ exist in gaseous wastes from industrial activities and automobile exhaust gas. Selective catalytic reduction (SCR) is the key industrial technology for NO_x_ removal by converting it to N_2_ with reducing gases (e.g., H_2_ and NH_3_) at high temperatures. Conventionally, metal oxides and molecular sieves ((M)_2_/nO·Al_2_O_3_·xSiO_2_·pH_2_O) are commonly used as supports to load active metals for NO_x_ SCR. However, the additional secondary metals (usually in the oxide form) tend to aggregate into large nanoparticles, decreasing the distribution of active sites and inhibiting metal–metal interactions for good NO_x_ reduction performance. 

**Figure 4:**
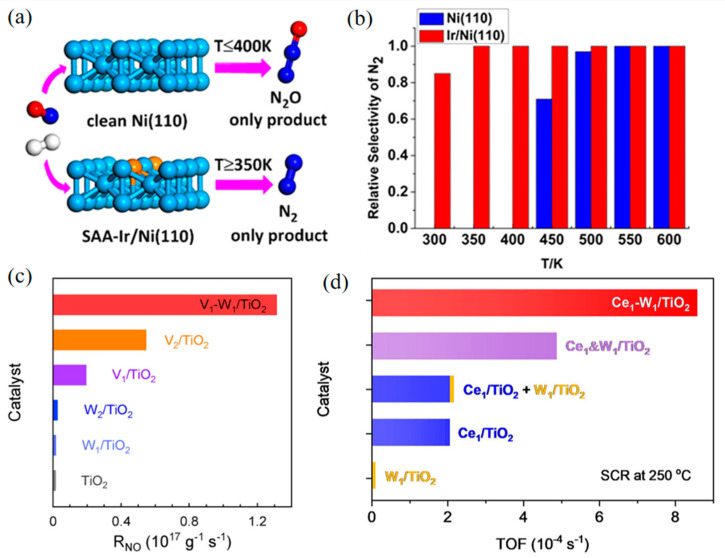
The single-atom alloy (SAA) Ir/Ni (110) promotes the reduction of NO into N_2_. (**a**) Mechanism diagram. (**b**) Relative selectivity of N_2_. Copyright 2019, American Chemical Society [69]. (**c**) Catalytic activities in terms of the reaction rates over the samples in SCR. Copyright 2022, Wiley-VCH [72]. (**d**) TOFs in SCR over Ce_1_&W_1_/TiO_2_, Ce_1_&W_1_/TiO_2_, Ce_1_/TiO_2_ + W_1_/TiO_2_, Ce_1_/TiO_2_, W_1_/TiO_2_, and TiO_2_ at 250 °C. Copyright 2022, American Chemical Society [73].

Therefore, in recent years, researchers have started to design bimetallic catalysts in the single-atom alloy (SAA) structure. In SAA, a small amount of an active metal is well distributed on the surface of another less active or less expensive metal to improve activity via enhancing metal–metal and metal–NO_x_ interactions. For example, Wen et al. investigated the reduction of NO with H_2_ on pure Ni and single-atom-Ir-doped Ni (Ir/Ni) surfaces by DFT calculations and microdynamics models [69]. The results showed that the doping of Ir greatly reduced the energy barrier of N_2_ generation and increased the energy barrier of N_2_O production (Figure 4a,b). In another study, a Cu–Pd dual-atom alloy (DAA) using Al_2_O_3_ as the support completely converted NO to N_2_ at 175 °C [70], with the N–O bond breaking of the (NO)_2_ dimer determined as the rate-limiting step. Single-atom Pd isolated by a large amount of Cu (Cu/Pd = 5) significantly improved the catalytic activity and N_2_ selectivity. After N–O bond breaking, N_2_O is decomposed into N_2_ smoothly on the Cu surface, which makes Cu and Cu-rich catalysts have high N_2_ selectivity. Both single-atom Pd and Cu active sites contribute to this highly efficient deNO_x_ system. 

Tang’s group has systematically designed several SAAs for NO_x_–SCR. A single-atom Mo_1_/Fe_2_O_3_ catalyst was synthesized for NO SCR [71], in which atomic Mo was anchored on reducible α-Fe_2_O_3_(001), thus a single-atom Mo ion and an adjacent Fe ion were constructed as a dinuclear site. In Mo_1_/Fe_2_O_3_, Mo ions provided Brønsted acid sites that converted to Lewis acid sites during SCR. This dinuclear structure showed high SCR TOFs comparable to V_2_O_5_/TiO_2_. Further, this group assembled single-atom V_1_ and W_1_ loaded on TiO_2_ (V_1_–W_1_/TiO_2_) [72], which realized tunable electronic interactions, thus performing significantly higher SCR rates (Figure 4c). Experimental and theoretical results indicated that the synergistic electron effect between V_1_ and W_1_ enriches high-energy spin charge around the Fermi level, enhancing the adsorption of reactant (NH_3_ or O_2_) and accelerating the surface reactions compared to individual V or W atoms. Besides, a dinuclear Ce_1_–W_1_/TiO_2_ catalyst was also developed to explore the synergistic effect between Ce and W in SCR [73]. The synergy of Ce_1_–W_1_ reduces the lowest unoccupied states of Ce_1_ near the Fermi level, boosting adsorption and oxidization of NH_3_, and renders the frontier orbital electrons of W_1_, speeding up O_2_ activation. Due to the strong electronic interaction within Ce_1_-W_1_ atom pairs, the TOF of Ce_1_–W_1_/TiO_2_ at 250 °C was four times higher than the sum of Ce_1_/TiO_2_ and W_1_/TiO_2_ (Figure 4d). 

With CO as the reducing agent, CO–SCR is regarded as a promising NO–SCR route because of its capacity to control two pollutant gases at the same time. However, the narrow reaction temperature window and the weak resistance to SO_2_ and O_2_ limit the application of CO–SCR. Ji et al. [74] developed a novel Ir SAC (IrW–WO_3_/KIT-6), with 1% Ir loaded on mesoporous SiO_2_ (KIT-6), and formed Ir–W intermetallic nanoparticles. At 250 °C and in the presence of 1% O_2_, NO was completely converted to N_2_ with 100% selectivity. At a wide temperature window (250–400 °C), the NO conversion rate of 80% and the N_2_ selectivity of 95% were achieved, better than those of Ir isolate-single-atomic-sites (Ir_1_–WO_3_/KIT-6) and Ir nanoparticles (Ir_n_–WO_3_/KIT-6); IrW–WO_3_/KIT-6 also showed excellent SO_2_ resistance. Furthermore, the team also developed a Pt SAC with negatively charged single-atom Pt (0.02 wt%) embedded on CuO squares and supported by CoAlO nanosheets (Pt−CuO/CoAlO) [75], showing 91% NO conversion and 80% N_2_ selectivity in 3% O_2_ at 200 °C. The interfacial electron transfer from CoAlO to CuO improved the electron density near Pt, thus enhancing NO adsorption, while Cu served as the adsorption site for CO. The Pt−CuO/CoAlO also showed no activity loss after 200 ppm SO_2_ heating for 15 h due to weakened SO_2_ adsorption on active sites.

The reaction between CO and NO also has implications for automobile exhaust treatment. SACs were also found to be efficient in emission control, typically in three-way catalysts (TWCs), achieving synergistic treatment of NO, CO, and hydrocarbons (HCs). Wang et al. [76] reported a dual-site catalyst composed of strongly coupled atomic Pt and Pd on CeO_2_, which was fabricated via a multi-step heating strategy. Compared with Pt SAC and Pd SAC, Pt–Pd SAC showed a lower T_90_ of NO and C_3_H_6_ conversion, while the T_90_ of CO oxidation was Pt–Pd SAC ≈ Pt SAC > Pd SAC.

**Figure 5:**
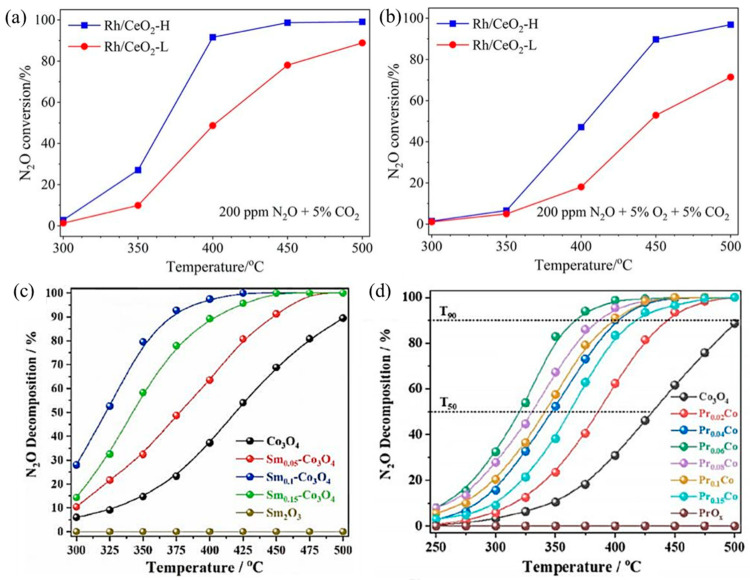
Catalytic performance for N_2_O decomposition over Rh/CeO_2_-L and Rh/CeO_2_-H catalysts under conditions of (**a**) 0.02% N_2_O + 5% CO_2_ and (**b**) 0.02% N_2_O + 5% O_2_ + 5% CO_2_; Ar balanced. Weight hour space velocity (WHSV) was fixed at 100,000 mL g^−1^·h^−1^. Copyright 2023, Chinese Society of Rare Earths [78]. (**c**) N_2_O decomposition activity normalized by specific surface area (S_BET_) on Co_3_O_4_ and Sm-doped Co_3_O_4_ samples. Copyright 2021, Elsevier B.V. [79]. (**d**) N_2_O decomposition activity normalized by S_BET_ on Co_3_O_4_ and Pr-doped Co_3_O_4_ samples. Copyright 2022, American Chemical Society [80].

N_2_O largely exists in the gas exhausts of nitric acid, adipate, and caprolactam industrial production and is also a byproduct during NH_3_–SCR to treat NO_x_. With an extremely high global warming potential (GWP) that is 298 times CO_2_-equivalent and 25 times CH_4_-equivalent, N_2_O is an important greenhouse gas [77]. For N_2_O direct decomposition (deN_2_O), it is generally decomposed at high temperatures (500–600 °C) by metal-loaded oxides or molecular sieves. In order to reduce the amount of noble metals, they are usually loaded on carriers with large specific surface areas, such as NiO, Co_3_O_4_, Al_2_O_3_, CeO_2_, and SiO_2_, to make them dispersed and improve deN_2_O activity. The catalytic performance of SACs depends largely on the coordination environment of metal sites. For example, Xie et al. obtained two different Rh_1_/CeO_2_ SACs with high and low coordination numbers (CN) by adjusting synthesis procedures [78]. The Rh_1_/CeO_2_ with higher Rh CN (Rh/CeO_2_-H) was more active in deN_2_O, which resulted from faster O_2_ desorption, more surface oxygen vacancies, and higher reducibility (Figure 5a,b). Li’s group loaded rare earth elements Sm [79] and Pr [80] onto Co_3_O_4_, respectively. By introducing Sm into Co_3_O_4_, the presence of Sm promoted the regeneration of the active site and improved the reducibility and oxygen desorption capacity of Co_3_O_4_. The catalytic performance of Sm_0.1_–Co_3_O_4_ showed ~52% N_2_O decomposition at 325 °C and over 90% N_2_O decomposition at 375 °C (Figure 5c). In addition, in Pr–Co_3_O_4_, the “Pr 4f–O 2p–Co 3d” network generated by Pr single-atom doping in Co_3_O_4_ redistributed electrons in the Co_3_O_4_ lattice, which greatly improved the N_2_O decomposition performance (Figure 5d). The T_50_ decreased from ∼430 °C of Co_3_O_4_ to ∼320 °C of Pr_0.06_Co, and the T_90_ decreased from ∼500 °C of Co_3_O_4_ to ∼367 °C of Pr_0.06_Co. 

### 2.4. CO_2_ Reduction

Electrochemical reduction of CO_2_ into various chemical feedstocks and fuels not only reduces the negative environmental impact of CO_2_ but also alleviates the problem of fossil fuel shortage [81,82,83]. In recent years, researchers have developed SACs as efficient catalysts for the electrochemical reduction of CO_2_ (CO_2_RR). Numerous heterogeneous catalysts, for example, metals [84,85,86], metal oxides [87,88], metal sulfides [89], metal organic frameworks (MOFs) [90], and their composites [91], have been used. In general, Ni and Fe SACs exhibited superior catalytic performance for CO evolution, while Co, Mn, and Zn SACs were relatively inert to CO_2_RR [92].

In a study by Zhang et al. [93], an isolated nickel monatomic electrode was prepared with high-density Ni(I) sites anchored to a nitrogen-doped carbon nanotube array and further encapsulated in a nickel–copper alloy on carbon fiber paper (Ni^I^–NCNT@Ni_9_Cu). The nickel–copper alloy was encased in the carbon-fiber paper. The combination of the single-atomic Ni(I) site and the self-supported array structure resulted in excellent CO_2_RR performance. The electron configuration of the *d* band of Ni was modified by introducing Cu, which enhanced the adsorption of hydrogen, thus hindering the hydrogen evolution reaction (HER). The specific current density of a single Ni atom electrode was 32.87 mA cm^−2^, with a TOF of 1962 h^−1^ at an overpotential of 620 mV and a Faradaic efficiency of 97% at around −0.73 V vs. RHE. Tang’s group [94] developed a Fe–N–C catalyst for CO_2_RR via a novel one-step calcination method, which achieved high selectivity of CO_2_RR to CO. Compared with pristine N–C material, Fe–N–C achieved a higher maximum Faradaic efficiency of 73% and a Tafel slope of 68 mV dec^−1^, respectively. The excellent CO_2_RR performance of the catalyst was ascribed to the active Fe–N_x_ sites, rich functional groups, and abundant microporous structure.

**Figure 6:**
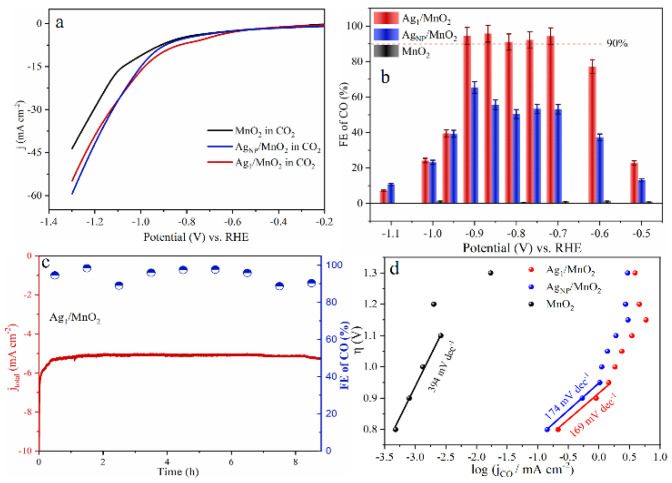
(**a**) Linear Sweep Voltammetry (LSV) curves of MnO_2_, AgNP/MnO_2_, and Ag_1_/MnO_2_ in a CO_2_-saturated 0.5 M KHCO_3_ electrolyte. (**b**) Faradaic efficiency of CO. (**c**) Long-term electrolysis experiments on Ag_1_/MnO_2_ at electrolysis potentials of −0.9 V vs. RHE. (**d**) Tafel plots of three samples. Copyright 2021, Angew Chem Int Ed Engl. [96].

As for noble metal catalysts, Au and Ag show relatively high CO_2_RR catalytic activity. It was shown that when comparing Ag and Au, when the NP size decreases, Au NPs will lead to the enhancement of the competitive HER, resulting in an increase in by-products, while Ag NPs can selectively enhance CO_2_RR [95]. Zhang et al. synthesized Ag_1_ monatomic catalyst (Ag_1_/MnO_2_) by thermal conversion of Ag NPs and surface reconstruction of MnO_2_ [96]. Ag_1_/MnO_2_ exhibited a Faradaic efficiency of 95.7% at −0.85 V vs. RHE (Figure 6b), with excellent stability in the reaction (Figure 6c). The Ag_1_/MnO_2_ showed improved CO_2_RR performance than conventional Ag nanocatalysts (AgNP/MnO_2_) and other reported Ag-based catalysts (Figure 6a,b,d). For current SACs in CO_2_RR, the low density of active sites, poor conductivity, and mass transfer resistance towards single atomic electrodes still limit their catalytic performance. In order to prevent metal aggregation on the cathode during reductive reactions and maintain the atomic dispersion, most current studies have only achieved a relatively low SAC metal loading below 5 wt%. Therefore, further research is still needed to improve the metal loading capacity of SACs.

## 3. Progress of SACs in Aqueous Pollution Control

### 3.1. H_2_O_2_-Based Fenton-like Processes

**Figure 7:**
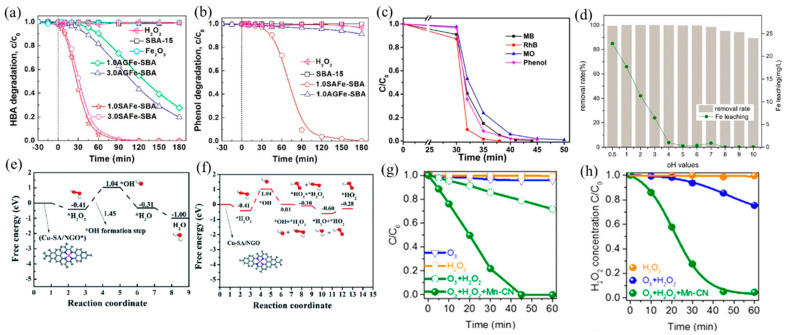
(**a**) HBA and (**b**) phenol adsorption and oxidation by H_2_O_2_ activation on catalysts. Copyright 2019, American Chemical Society [97]. (**c**) Removal efficiency of different organic pollutants using an I–FeN_x_/g–C_3_N_4_-5 catalyst [28]. Reaction conditions: 200 mg L^−1^ organics (MB, MO, RhB, and phenol), 77 mM H_2_O_2_, 0.5 g L^−1^ catalyst, 308 K, and visible light. Copyright 2018, American Chemical Society. (**d**) Effect of pH values on AR 73 removal and Fe leaching. Copyright 2016, Elsevier B.V. [98]. Free energy diagrams for Cu–SA/NGO in ^•^OH generation under acidic (**e**) and neutral (**f**) conditions. Copyright 2020, The Royal Society of Chemistry [26]. (**g**) Degradation curves of OA in ozonation, the H_2_O_2_ process, and the peroxone process with or without Mn–CN. (**h**) Corresponding H_2_O_2_ decay curves in the H_2_O_2_ process, peroxone process, and peroxone process with Mn–CN. Copyright 2019, American Chemical Society [99].

Fenton process with H_2_O_2_ generates strongly oxidizing ^•^OH for aqueous organic pollutant decomposition. The traditional Fe-based catalysts have been reported the most, among which Fe SACs have a better catalytic ability than nanoparticle catalysts. Yin et al. [97] reported a SAFe–SBA catalyst with single-atom Fe dispersed into the nanopores of SBA-15. The well-dispersed Fe atoms promoted the decomposition of H_2_O_2_ into ^•^OH, leading to a better catalytic performance of SAFe–SBA than aggregated iron sites (AGFe–SBA). The degradation efficiency of both HBA and phenol reached 100% after 180 min (Figure 7a,b). In addition, the Fe SACs formed by Fe sites embedded in g–C_3_N_4_ effectively degraded a variety of dyes and organic pollutants (methylene blue (MB), methyl orange (MO), rhodamine B (RhB), and phenol) (Figure 7c), owing to the improved production of ^•^OH from H_2_O_2_ activated by Fe(II)–N_x_ active sites [28]. 

The narrow pH range (2–4) hinders the real application of the Fenton processes [100], which can be improved by adjusting the support and coordination environment of the catalyst. Ma et al. [98] found that dispersing SA Fe–g–C_3_N_4_ onto graphitized mesoporous carbon composite (GMC) broadened the working pH window. The obtained catalyst exhibited high catalytic activity in the range of pH = 4–10, attributable to the well-dispersed Fe–N_x_ and *π*–*π* stacking of GMC that promoted the adsorption and decomposition of H_2_O_2_ (Figure 7d). Besides, Wu and coworkers [26] prepared a high density of Cu SACs on N-doped graphene (Cu–SA/NGO), which also achieved efficient H_2_O_2_ decomposition at neutral pH facilitated by Cu–N_4_ active sites and low energy barriers of reaction (Figure 7e,f). At acidic conditions, H_2_O_2_ can easily be adsorbed on Cu–N_4_ sites and generate OH* and ^•^OH, while at neutral conditions, OH* s formed when adsorbed H_2_O_2_ reacted with another H_2_O_2_ molecule to form oxidative HO_2_*. Gong and coworkers [99] developed Mn–N_4_-doped g–C_3_N_4_ (Mn–CN), which catalyzed the formation of ^•^OH with H_2_O_2_ and additional oxidant O_3_ and degraded oxalic acid (OA). Because of the dispersion of isolated Mn atoms, Mn–CN showed excellent catalytic performance, and oxalic acid was completely degraded within 45 min (Figure 7g,h). Different from the traditional H_2_O_2_ reaction, this work proposed a new pathway: H_2_O_2_ adsorbed on Mn–N_4_ sites formed HOO–Mn–N_4_ species, which reacted with O_3_ to generate HO_2_^•^ and O_3_^•−^, finally producing ^•^OH.

### 3.2. Persulfate-Based Fenton-like Processes

In recent years, persulfate-based AOPs have been widely applied in water purification, which is mainly based on the chain reactions initiated by persulfate (PMS, PDS) molecules, generating strongly oxidizing ROS including SO_4_^•−^, ^•^OH, O_2_^•−^, and ^1^O_2_ [101,102]. The process has a strong oxidation capacity and a wide range of solutions for environmental adaptation. In persulfate-AOPs, Co-based, Fe-based, Cu-based, and Mn-based catalysts are widely studied.

**Figure 8:**
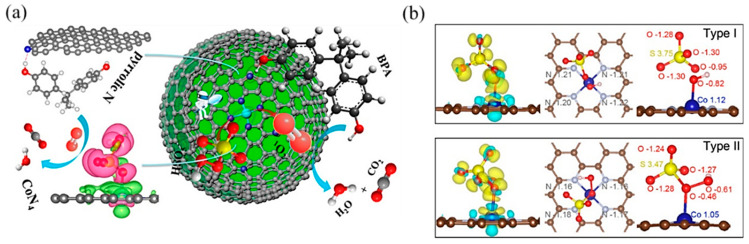
(**a**) The proposed overall Fenton-like reaction mechanism on a single-Co-atom catalyst. Copyright 2018, American Chemical Society [103]. (**b**) Adsorption configuration and charge density of PMS on Co–TPML through coordination with H-adjacent (Type I) and S-adjacent (Type II) O atoms in the peroxide bond, respectively. Yellow and cyan denote the electron accumulation and electron depletion, respectively. Copyright 2020, American Chemical Society [104].

In PMS-based AOPs, Co-based SACs have been extensively studied [101]. Single Co atoms anchored onto porous N-doped graphene showed dual reaction sites [103]: the Co atom was the reaction site, while the adjacent pyrrolic N was the adsorption site (Figure 8a). It activated PMS to degrade BPA with high efficiency because the dual reactive sites reduced the transport distance of ROS and improved the mass transfer efficiency. Likewise, Kim’s group [104] reported pyridine N-coordinated single-atom Co loaded on a polyromantic macrostructure (Co–TPML) (Figure 8b), which also showed outstanding PMS activation and achieved high pollutant removal efficiency, resulting from a high-density and ultrafine dispersion of Co single atoms. With beneficial π-conjugation of TPML and strong metal–support interactions, peroxide adsorption and activation were enhanced. Furthermore, this group developed a single-atom Co-loaded 2D Graphene Oxide (GO)-based membrane [105], in which vitamin C was applied as a mild reducing agent to improve the atomically Co dispersion and maintain the structure of GO layers. This study observed that the Co_1_–GO membrane showed excellent ability for 1,4-dioxane degradation with the addition of PMS. The kinetics of 1,4-dioxane degradation were over 640 times greater than those in suspension, which was the highest among reported studies in persulfate-based 1,4-dioxane degradation. This catalyst–membrane combination was able to repel macromolecular organic matter, reducing its scavenging effect on free radicals. In addition, studies have found that the porous carbon material support can promote electron transfer [106], which is conducive to improving the efficiency of PMS-based AOPs.

Compared with PMS, PDS is more difficult to activate with a short peroxide O–O bond (1.322 Å) in the structure of –O_3_S–O–O–SO_3_– [107]. Because of its cheaper cost, lower toxicity, and lower pH limits, PDS is expected to be more widely applied in actual water treatment. Generally, in PDS/SAC systems, synergistic effects between the atomic metal and the support play an important role [15]. Li and coworkers [108] developed Cu single sites dispersed on carbon nitride (SAS–Cu_1.0_), showing remarkable performance in tetracycline degradation due to the enhanced PDS adsorption and activation. Under UV light and 0.1 mM sodium persulfate, the tetracycline (TC) degradation rate of SAS-Cu_1.0_ reached 82.5% in 30 min, while the degradation rates of carbon nitride (CN) and CN–NanoCu were 53.5% and 78.1%, respectively. The result revealed that the degradation mechanism on single-atom Cu involved both radical and nonradical pathways, leading to the promotion of charge separation and transfer. 

### 3.3. Electrocatalytic Hydrodehalogenation 

Organic halides that contain C–X bonds (X = Cl, Br, I, and F), such as chlorobenzene, 4-chlorophenol, and bromophenol, are commonly found in water bodies contaminated by pharmaceuticals, pesticides, surfactants, and after disinfection by chlorine [109]. Due to their strong carbon–halogen bonds and the ability to destruct biological enzymes, organohalogens are difficult to destroy by biological methods, leading to their persistent existence in water and posing a serious threat to human health, the ecological environment, and agricultural production. To solve this problem, hydrodehalogenation is proposed as an effective dehalogenation scheme in which two distinct partially charged H^δ−^ and H^δ+^ atoms formed from H atoms are utilized to attack carbon–halogen bonds [110,111]. Direct catalytic hydrodehalogenation is the most studied dehalogenation method at present. Numerous mono-metal and bimetallic catalysts have been developed. However, high catalyst costs, strict reaction conditions, and unsatisfactory catalytic efficiency make this technology a dilemma. In comparison, electrocatalytic and photocatalytic hydrodehalogenation, which are environmentally friendly and energy saving, have become a hot research field in recent years. In 1975, Geer et al.’s experiment on hydrodehalogenation of hexachlorobezene (HCB) by electrocatalysis proved that the complete degradation of chlorinated organic compounds could be achieved by controlling the potential [112]. 

**Figure 9:**
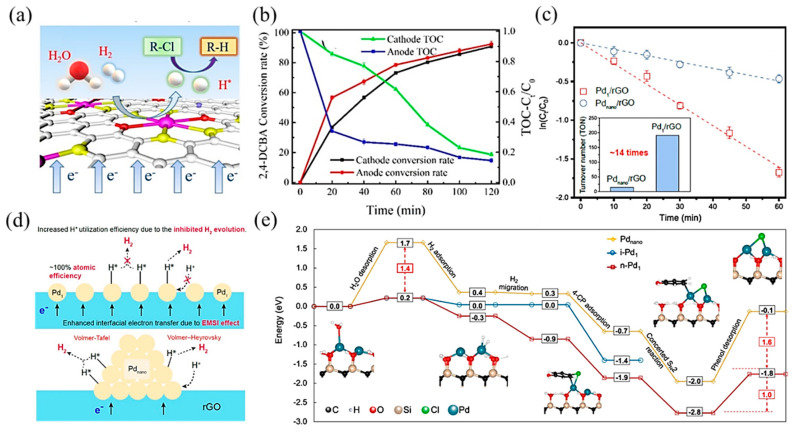
(**a**) Schematic illustration of the electrocatalytic hydrodechlorination reaction mechanism. (**b**) Total Organic Carbon (TOC) concentration ratio and conversion rate of 2,4-DCBA over time. Copyright 2020, American Chemical Society [113]. (**c**) Pseudo-first-order kinetic plots of 4-CP dechlorination with Pd_1_/rGO and Pd_nano_/rGO electrodes. The inset indicates the turnover number per Pd atom based on a reaction time of 30 min. (**d**) Proposed mechanism of enhanced cathodic hydrodechlorination with Pd_1_/rGO versus Pd_nano_/rGO. Copyright 2021, American Chemical Society [115]. (**e**) Hydrodehalogenation on i-Pd_1_, n-Pd_1_, and Pd_nano_. The blue, black, red, tan, and white spheres in geometrical models are Pd, C, O, Si, and H atoms, respectively. The solid and dashed lines represent the minimum energy path and other reaction pathways, respectively. Copyright 2021, Springer Nature [116].

Transition metal-based SACs have been used due to their excellent electrocatalytic hydrodehalogenation performance. Wang et al. [113] synthesized single-atom Co on sulfide graphene (Co–SG), achieving high atomic H* production by electrochemical reduction of H_2_O and electrolysis of hydrogen. With the synergistic effects among Co active sites, S-doped graphene, and the interfacial structure, the conversion rate of 2,4-DCBA reached 91.1% and the TOC concentration was reduced by 80% (Figure 9a,b). Zhao and coworkers [114] developed a Fe/Cu bimetallic single-atom catalyst dispersed on N-dope porous carbon (FeCuSA–NPC), leading to a stronger chlorinated pollutant degradation effect. In this process, dichlorination on the Cu single atom and hydroxyl radical oxidation on the Fe single atom formed a synergistic effect, which led to a high removal activity for 3-chlorophenol (3-CP), 2,4-dichlorophenol (2,4-DCP), and 2,4,6-trichlorophenol (2,4,6-TCP), with kinetics between 545.1 and 1374 min^−1^ g_metal_^−1^. 

Apart from transition metal catalysts, noble metal catalysts are also efficient in electrocatalytic hydrodehalogenation, among which, Pd-based catalysts have been studied in depth. Huang et al. [115] synthesized a single-atom Pd loaded on reduced graphene oxide (Pd_1_/rGO), which was more effective in chlorinated phenol dichlorination and showed higher atomic efficiency than Pd nanoparticle counterparts (Figure 9c). Mechanistic studies showed that this promotion effect was attributed to two aspects: (1) a strong interaction between the metal and support enhanced interfacial electron transfer through Pd–O bonds; (2) Pd_1_ restrained H_2_ evolution, contributing to atomic H (H*) utilization (Figure 9d). Further, Chu et al. [116] proposed that neighboring Pd single-atom catalysts, with shorter distances and more adjacent active sites between atoms, performed higher activity and selectivity in hydrogenating carbon–halogen bonds than isolated single-atom Pd. DFT calculations (Figure 9e) revealed that the cooperative effect between neighboring Pd atoms decreased the energies of water desorption and hydrogenated product desorption, which were the key meta-stable reaction steps. Besides, the neighboring structure was conducive to selectively hydrogenating the C–Cl bond without affecting the other bonds. 

### 3.4. Photocatalytic Hydrodehalogenation

Photocatalytic hydrodehalogenation realizes the fracture of carbon–halogen bonds through photoexcitation and electron transfer. Numerous studies have proved that semiconductor catalysts doped with noble metals display superior photocatalytic hydrodehalogenation.

**Figure 10:**
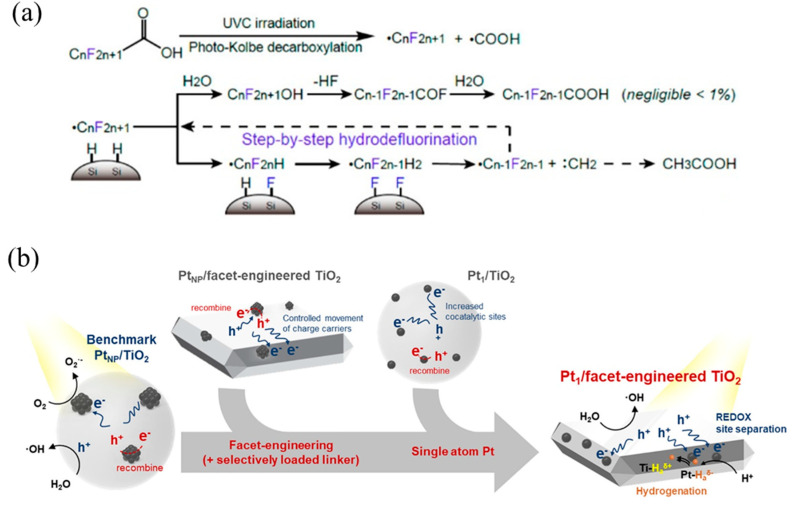
(**a**) PFOA decomposition mechanism with Pt_1_/SiC showing photo-Kolbe decarboxylation and subsequent hydrodefluorination. Copyright 2018, American Chemical Society [29]. (**b**) Photocatalysis mechanisms of Pt nanoparticle-loaded TiO_2_, Pt single-atom-loaded TiO_2_, Pt nanoparticle-loaded facet-engineered TiO_2_, and Pt single-atom-loaded facet-engineered TiO_2_. Copyright 2021, American Chemical Society [117].

Kim’s team has reported single-atom Pt supported on SiC (Pt_1_/SiC) [29] and TiO_2_ (Pt_1_/TiO_2_) [117], respectively, achieving hydrodehalogenation of perfluorooctanoic acid (PFOA) by cleaving C–F bonds. As for Pt/SiC (Figure 10a), due to the high work function of Pt (~5.65 eV), it tended to attract photogenerated electrons from the SiC conduction band, and then H atoms were selectively reduced and formed Pt–H bonds through the Volmer reaction. Finally, H atoms spillover from Pt–H bonds were transferred to SiC to form Si–H, which was then redistributed with the C–F bond, thus achieving hydrodehalogenation. Likewise, Pt single atoms in Pt/TiO_2_ drove the photogenerated electrons on the conduction band to generate H atoms and spill over onto the TiO_2_ surface, further forming Ti–H bonds to break C–F (Figure 10b). On the contrary, Pt nanoparticles consumed photogenerated electrons to reduce O_2_, instead of hydrodehalogenation.

In addition, single-atom Ag was confirmed as an ideal catalyst to selectively dehalogenate under visible-light irradiation by Wang et al. [118]. Under mild visible light irradiation, AgF was successfully reduced to Ag(0) single atoms and Ag nanoparticles. Theoretical and experimental investigations suggested that such mixed species (MS-Ag) showed outstanding hydrodehalogenation and deiodination-arylation performance, resulting from the synergistic effects of the Ag single atoms and the light-harvesting unit of Ag nanoparticles. Notably, the yield of selective hydrodehalogenation of 4-iodoanisole was up to 99% when CsF was added.

### 3.5. Nitrate and Nitrite Reduction

Nitrate (NO_3_^−^) and nitrite (NO_2_^−^) are common inorganic nitrogen-containing pollutants in the aqueous phase and the main causes of eutrophication and algae blooms. Due to the excessive use of agricultural fertilizers and improper treatment of sewage, NO_3_^−^ is prevalent in groundwater and surface water bodies, posing a great threat to human health and the environment. NO_3_^−^ in sewage can be converted into NO_2_^−^ by microorganisms, which will destroy the oxygen transport ability of hemoglobin when entering the human body, and even lead to poisoning or cancer. Nitrate reduction reaction (NO_3_RR) is a promising strategy to reduce the environmental pollution caused by NO_3_^−^, while producing N_2_ or NH_3_ as a valuable energy source. 

**Figure 11:**
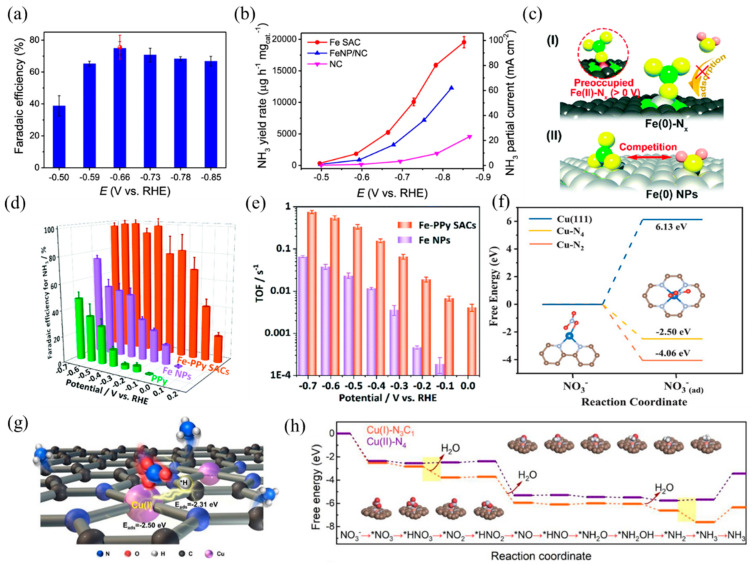
(**a**) NH_3_ Faradaic efficiency of Fe SAC at each given potential. Red dot is Faradaic efficiency estimated by three independent NMR tests. (**b**) NH_3_ yield rate and partial current density of Fe SAC, FeNP/NC, and NC. Copyright 2021, Springer Nature [30]. (**c**) The proposed preoccupied NO_3_RR mechanism for the single-site center (I) and the classical competitive mechanism for the bulk surface (II). (**d**) Faradaic efficiency for ammonia and (**e**) TOFs of the Fe–PPy SACs and Fe NPs based on the result of SI-SECM for ammonia production. Copyright 2021, The Royal Society of Chemistry [119]. (**f**) Calculated free energies for NO_3_^−^ adsorption on Cu (111), Cu–N_4_, and Cu–N_2_ surfaces, respectively. The brown, gray, blue, and red balls represent C, N, Cu, and O atoms, respectively. Copyright 2020 Wiley-VCH [120]. (**g**) TOC and (**h**) calculated activation energy for NO_3_RR using Cu(I)–N_3_C_1_ and Cu(II)–N_4_ as models. Copyright 2022, American Chemical Society [121].

Single-atom electrocatalysts can realize efficient NO_3_RR and selectively obtain NH_3_, such as Fe- and Cu-based SACs. Primarily, the active center of the Fe-based catalyst is Fe–N_x_. According to Wang et al. [30], isolated Fe single atoms in the form of Fe–N_4_ hindered N–N coupling, resulting in higher affinity towards N–H coupling and NH_3_ formation. Benefiting from these structure advantages, the nitrogen-coordinated Fe sites dispersed on carbon matrix exhibited remarkable capacities in NO_3_RR with a Faradaic efficiency of ~75% and a high NH_3_ yield of ~20,000 μg h^−1^ mg_cat._^−1^ (Figure 11a,b). In addition, Liu et al. [31] prepared a highly active and selective Fe–CNS consisting of Fe single atoms loaded on S and N-doped carbon supports. S-doping created more defects on the support surface, which was beneficial to enhancing the stability of Fe single atoms. Along with Fe–N_4_, the presence of S sites adjusted the coordination environment and formed FeN_4_S_2_ as the dominant active site. The experimental results of NO_3_RR revealed that the prepared Fe–CNS catalyst performed excellent activity with a nitrate removal capacity of 7822 mg-N g^−1^ Fe and a high ammonia Faradaic efficiency of 78.4%. Yu et al. [119] found that nitrate preoccupied on Fe(II)–N_x_ and hindered the adsorption of H_2_O, thus inhibiting the competitive reaction of HER. In addition, the special thermodynamic and kinetic properties of the Fe SACs resulted in a more positive and narrower range of redox potentials than the Fe NPs (Figure 11c). As a result, Fe SACs achieved a higher NH_3_ yield and selectivity, with a maximum yield rate of 2.75 mg_NH3_ h^−1^ cm^−2^ and close to 100% Faradaic efficiency (Figure 11d). The TOFs of the Fe–PPy SACs reached 0.006–0.7 s^−1^ at 0–−0.7 V vs. RHE, while the TOFs of the Fe NPs were 0.00015–0.06 s^−1^ (Figure 11e). 

Besides, Cu-based SACs with the Cu–N_x_ coordination structure are also suitable for NO_3_RR, according to previous studies. Feng et al. [120] reported a Cu SAC anchored on nitrogen-doped carbon nanosheets (Cu–N–C) with high activity, selectivity, and stability in NO_3_RR. XAFS analysis and DFT calculations revealed that the mixed coordination structures of Cu–N_2_ and Cu–N_4_ dispersed on carbon caused the adsorption of NO_3_^−^ and NO_2_^−^ (Figure 11f), inhibiting the release of NO_2_^−^. At −1.3 V vs. SCE with an initial 50 mg L^−1^ NO_3_-N, the selectivity of the NO_2_-N product was only 5%. Fan and coworkers [121] studied the NO_3_RR properties of atomic Cu supported on micro/mesoporous nitrogen-doped carbon (Cu MNC). The Cu(I) sites (Cu(I)–N_3_C_1_) concentrated the charge around the center Cu atoms, causing the adsorption of *NO_3_ and *H to adjacent Cu and C sites by balanced adsorption energy. Compared with Cu(II)–N_4_, Cu(I)–N_3_C_1_ decreased the activation energy of rate-limiting steps, thus promoting the formation of NH_3_ (Figure 11g,h). When applied to nitrate reduction (100 mg-N L^−1^), Cu MNC achieved a promising NH_3_ yield rate per active site of 5466 mmol g_Cu_^−1^ h^−1^ and a conversion rate of 94.8% within 6 h.

As for noble metal SACs, isolated Ru sites dispersed on nitrogen-doped carbon (Ru SA–NC) were demonstrated to be an effective catalyst for both nitrate and nitrite electroreduction to NH_3_ [122]. Ru SA–NC achieved Faradaic efficiencies of 97.8% at −0.6 V vs. RHE (NO_2_^−^ reduction) and 72.8% at −0.4 V vs. RHE (NO_3_^−^ reduction), respectively. A bimetallic catalyst with a single-atom Ru-modified Cu nanowire array loaded on Cu foam (Ru–Cu NW/CF) was proposed by Lee’s group [123], which showed efficient electrocatalytic nitrite reduction. Due to the inhibition of N–N coupling by the active site of single-atom Ru, at the overpotential of −0.6 V vs. RHE, the Faradaic efficiency reached 94.1% and the NH_3_ yield was up to 211.73 mg h^−1^ cm^−2^. Kamiya et al. [124] prepared an atomically dispersed Pt-modified covalent triazine framework hybridized with carbon nanoparticles (Pt–CTF/CP), which showed a NO_2_^−^ reduction activity comparable to that of bulk Pt surfaces. It is worth noting that nitrate reduction reactions were almost not detectable. Since nitrate adsorbed on the single Pt atom is in an unstable monodentate form, the nitrate may not have enough adsorption energy to be activated.

## 4. Conclusions and Outlook

With close to 100% atomic efficiency and high catalytic activity, SACs are considered to bring new opportunities for environmental pollution remediation and are becoming a prevalent research frontier. The well-controlled atomically dispersed structure of SACs fills the gap between heterogeneous and homogeneous catalytic reactions and provides a new direction for understanding the catalytic mechanism at the atomic level. Compared with bulk NPs, SACs’ unique electronic characteristics and atomic sites help them achieve reactions that cannot be catalyzed by NPs. Additionally, with higher atomic utilization and less metal loading, SACs have a lower cost of raw materials, showing economic advantages in practical engineered applications. In the past decade, researchers have developed a variety of SACs that have been successfully applied to solve practical environmental problems, such as the purification of industrial gaseous pollutants and the treatment of organic pollutants in wastewater. In general, SACs show excellent catalytic activity, selectivity, and stability in various catalytic reactions.

However, current SACs still have non-negligible shortcomings that should be overcome. Due to ultra-low metal loading, the catalytic efficiency of SACs is unsatisfactory. To improve the reaction efficiency, it is a tough challenge to avoid single-atom aggregation when increasing the metal content. From the above discussions, we found that the coordination structure and interactions between the atomic metal and the support have an important impact on the physicochemical properties and catalytic performances of SACs. Nevertheless, there is still a lack of clarity on the structure–catalytic correlation. Besides, unlike laboratory experiments, complex compositions in the actual gas or water bodies might interfere with the catalytic reaction via surface contamination and deactivation of the catalyst. A long-term reaction may also lead to the loss of metal atoms or aggregation. In engineering applications, the integration of SACs into existing devices or systems is also an important issue. To promote further development of SACs in environmental engineering, the following research directions are proposed:

**(1)**  **Development of new synthetic strategies:** Increasing the number and density of coordination sites can effectively improve the loading of metal single atoms. More loading sites can be created by fabricating defects and unsaturated coordination centers. The methods for synthesizing stable SACs with relatively high metal loadings should be further developed. Studies revealed that when the SAC content increases from ~1% to ~5%, monatomic metals will form neighboring SACs or SAC ensembles without metal−metal bonding. However, it still maintains high atomic utilization and a unique coordination environment [101]. Recently, atom-trapping methods have been applied to load 1–3 wt% of SACs onto reducible supports (e.g., CeO_2_, FeO_x_), preventing metal aggregation at high temperatures [17]. It was demonstrated that a single-atom Cu catalyst prepared by atom-trapping on CeO_2_ effectively prevented sintering and deactivation via the regulated charge state of the Cu through facile charge transfer between the active site and the support [125]. Moreover, using graphene quantum dots as the carbon carrier, the transition metal SAC content was further increased to nearly 40% [126]. Appropriate supports, such as porous carbon and MOF, can strengthen metal–substrate interactions. In addition, it is important to develop a synthetic strategy that can precisely regulate the atomic active center and create more selective metal active centers for a specific catalytic reaction. Through doping heteroatoms and designing bimetallic sites, creating synergistic interactions between various elements may greatly contribute to the enhancement of SAC performance.
**(2)**  **Study on catalytic mechanisms:** At present, most of the characterization techniques are ex situ, such as high-angle annular dark-field–scanning transmission electron microscopy (HAADF–STEM) and X-ray absorption spectroscopy (XAS), which make it difficult to provide in situ characterization of the alterations of the physicochemical properties and electronic structures of SACs during the reactions. Hence, it is necessary to develop advanced in situ characterization technology to further study the complex pathways of catalytic reactions at the atomic level. Nowadays, some cutting-edge in situ characterization techniques have been reported to detect the evolution of catalyst sites and the interactions between active sites and reactants during the reaction process. For example, Hensen et al. [59] used an in situ near ambient pressure X-ray photoelectron spectrometer (NAP–XPS) to follow the surface electronic structure of Pd–CeO_2_ SAC during CO oxidation and in situ infrared spectroscopy to probe the interaction between surface sites and reactants. Thereby, the structure–function relationships of Pd/CeO_2_ catalysts were established. In addition, in situ and operando infrared and XAS were used to detect CO oxidation mechanisms on an Ir single atom, detailing reaction steps [127]. Datye et al. [128] also used CO as a probe molecule during in situ DRIFTS to effectively detect the property changes of Pt_1_/CeO_2_ under reaction conditions. The model establishment and theoretical calculations by DFT are beneficial to understanding the formation of the intermediate products and energy barriers (i.e., the rate-determining step) during the reaction, which can guide the design of future catalysts. However, when faced with complicated environmental media and operating parameters, DFT is not suitable due to the high cost of time. As a more handy and advanced technology, machine learning (ML) and quantitative structure–activity relationship (QASR) can efficiently establish the relationship between catalyst performance and certain specific descriptors, such as operational parameters.
**(3)**  **Optimization for practical applications:** To stabilize the interactions between metal atoms and support, the synthesis methods of a certain metal–support combination are specific, which may hinder the large-scale synthesis of SACs. Developing a simple and general synthesis strategy is beneficial to reducing the cost of large-scale SAC production. The integration of SACs into reactors or systems to achieve pilot-scale and large-scale is another troublesome challenge to overcome. Besides, it is of great importance to improve the adaptability to different complex environments and the stability of the reaction system.

## Data Availability

No new data were created or analyzed in this study. Data sharing is not applicable to this article.
